# Outbreaks of Cyclosporiasis — United States, June–August 2013

**Published:** 2013-11-01

**Authors:** M. desVignes-Kendrick, Kaye Reynolds, Teresa Lee, Linda Gaul, Kate Klein, Kari Irvin, Allison Wellman, Angela Hardin, Ian Williams, Ryan Wiegand, Julie Harris, Monica Parise, Francisca Abanyie, R. Reid Harvey

**Affiliations:** Fort Bend County Health and Human Svcs; City of Rosenberg Health Dept; Texas Dept of State Health Svcs; Coordinated Outbreak Response and Evaluation Network, Office of Foods and Veterinary Medicine, Food and Drug Administration; Div of Foodborne, Waterborne, and Environmental Diseases, National Center for Emerging and Zoonotic Infectious Diseases; Div of Parasitic Diseases and Malaria, Center for Global Health; EIS officers, CDC

During June–August 2013, CDC, state and local public health officials, and the Food and Drug Administration (FDA) investigated an unusually large number of reports of cyclosporiasis (compared with annual reports to the National Notifiable Disease Surveillance System [e.g., 123 cases in 2012]), an intestinal infection caused by the parasite *Cyclospora cayetanensis* (1). By September 20, CDC had been notified of 643 cases from 25 states, primarily Texas (278 cases), Iowa (153), and Nebraska (86). Investigations in Iowa and Nebraska showed that restaurant-associated cases in these two states were linked to a salad mix that contained iceberg lettuce, romaine lettuce, red cabbage, and carrots (2). Most patients in Iowa and Nebraska became ill during June 15–29; cases reported during July and August were primarily from Texas (Figure).

CDC collaborated with state and local public health officials in Texas and the FDA to investigate a cluster of illnesses among patrons of a Mexican-style restaurant in Fort Bend County, Texas (restaurant A). A case of restaurant A–associated gastroenteritis was defined as gastrointestinal illness in a person who had eaten at restaurant A after June 1, 2013. Of 30 persons who ate at restaurant A, 22 had laboratory-confirmed *C. cayetanensis* infections, and eight had no laboratory confirmation. To identify the source or sources of the infections, a case-control study using 21 case-patients (15 laboratory-confirmed and six probable) with known meal dates and 65 controls matched by restaurant A meal date was conducted.

Case-patients and controls were asked about the meals they ate at restaurant A, using the menu. Ingredient-level analyses were conducted using meal consumption data and restaurant A recipes to identify four fresh produce ingredients with a statistically significant association with illness: fresh cilantro (matched odds ratio [mOR] = 19.8; 95% confidence interval [CI] = 4.0–>999), whole onions (mOR = 15.3; CI = 2.1–697.7), garlic (mOR = 10.7; CI = 1.5–475.4), and tomatoes (mOR = 5.5; CI = 1.1–54.1). Only fresh cilantro was consumed by all case-patients included in the study. In addition, of the four restaurant-produced salsas served at restaurant A, three containing fresh, uncooked cilantro were associated with illness: hot salsa (mOR 8.0; CI = 2.3–31.4), side salsa (mOR 5.7; CI = 1.6–23.7), and fire salsa (mOR 3.5, CI = 1.1–12.7). Case-patients also more commonly than controls reported eating salsa ranchera, which contained fresh cooked cilantro, but the association was not statistically significant: (mOR = 6.0; CI = 0.7–75.2).

Traceback information indicated that Puebla, Mexico, was a source of fresh cilantro served to ill persons at restaurant A. Lettuce served at restaurant A was neither sourced from the same producer implicated in the outbreak investigation in Iowa and Nebraska nor was it associated with illness. Additionally, restaurant A did not use red cabbage or carrots. Taken together, data from tracebacks and epidemiologic investigations in Texas, Iowa, and Nebraska indicate that more than one outbreak of cyclosporiasis occurred during summer 2013 in the United States, and that the food item associated with illness in Texas was different from that implicated in restaurant-associated cases in Iowa and Nebraska.

## Figures and Tables

**FIGURE:**
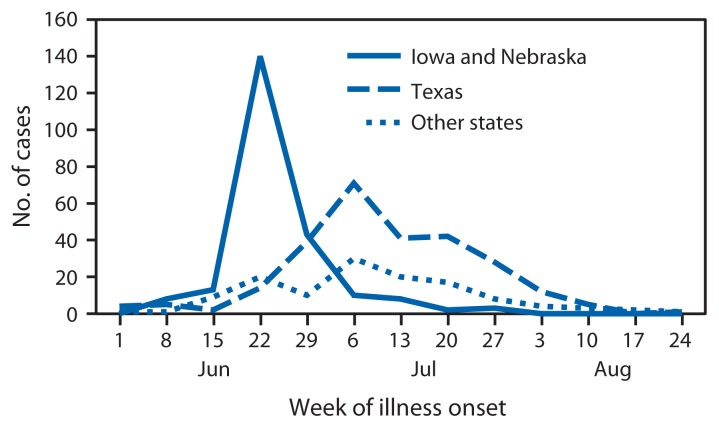
Laboratory-confirmed cyclosporiasis cases by week of onset — United States, June 1–September 10, 2013
